# Pattern Formation in a Spatially Extended Model of Pacemaker Dynamics in Smooth Muscle Cells

**DOI:** 10.1007/s11538-022-01043-1

**Published:** 2022-07-08

**Authors:** H. O. Fatoyinbo, R. G. Brown, D. J. W. Simpson, B. van Brunt

**Affiliations:** School of Mathematical and Computational Sciences, Massey University, Palmerston North, New Zealand

**Keywords:** Pattern formation, Smooth muscle cells, Pacemaker dynamics, Morris–Lecar, Bifurcation analysis, Non-Turing patterns, Spatiotemporal chaos, Travelling waves, 37N25, 74H60, 37M20, 37C75, 92C30

## Abstract

Spatiotemporal patterns are common in biological systems. For electrically coupled cells, previous studies of pattern formation have mainly used applied current as the primary bifurcation parameter. The purpose of this paper is to show that applied current is not needed to generate spatiotemporal patterns for smooth muscle cells. The patterns can be generated solely by external mechanical stimulation (transmural pressure). To do this we study a reaction-diffusion system involving the Morris–Lecar equations and observe a wide range of spatiotemporal patterns for different values of the model parameters. Some aspects of these patterns are explained via a bifurcation analysis of the system without coupling — in particular Type I and Type II excitability both occur. We show the patterns are not due to a Turing instability and that the spatially extended model exhibits spatiotemporal chaos. We also use travelling wave coordinates to analyse travelling waves.

## Introduction

Smooth muscle cells (SMCs) can be found throughout the body. They are present, for example, in blood vessels, and provide a variety of essential functions. The contraction and relaxation of SMCs regulate organ function, such as the blood flow rate in blood vessels (Lamboley et al. 2003; Shaikh et al. 2011). SMCs aid in digestion and nutrient collection in the gastrointestinal tract (Bitar 2003; Harnett et al. 2005), and regulate bronchiole diameter in the respiratory system (Chung 2000). In the urinary system, they play a role in removing toxins and in electrolyte balance (Alexander 1973; Andersson and Arner 2004). Like other excitable cells (e.g. neuron, endocrine, and skeletal cells), when stimulated SMCs can generate a large electrical signal (action potential) and contract in response. This process is known as electro-mechanical coupling.

**Fig. 1:**
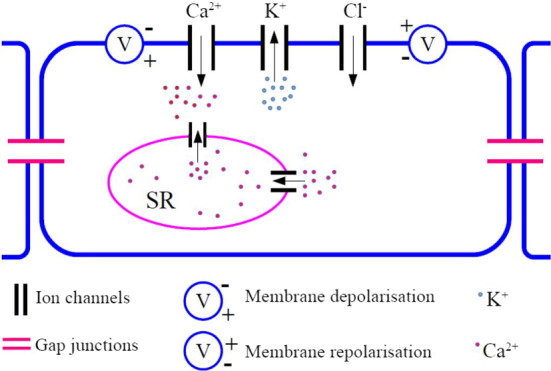
A schematic representation of electrically coupled smooth muscle cells. The concentration gradient of $\mathrm{Ca}^{2+}$ between the inside and the outside of the cell results in fluxes of ions into the cell through the $\mathrm{Ca}^{2+}$ channel and then into the sacroplasmic reticulum (SR). During this process the membrane potential becomes greater than the resting potential, hence the membrane is depolarised. The depletion of $\mathrm{Ca}^{2+}$ in the sacroplasmic reticulum induces an efflux of $\mathrm{K}^{+}$ through the $\mathrm{K}^{+}$ channel which causes the membrane potential to return to its resting state. The leak ($\mathrm{Cl}^{-}$) channel is a non-selective channel that is always open for ion passage (Color figure online)

Electro-mechanical coupling in the cell membrane of a SMC is mediated by the influx of extracellular $\text {Ca}^{2+}$ through voltage-gated $\text {Ca}^{2+}$ channels and $\text {Ca}^{2+}$ release from the cell’s internal $\text {Ca}^{2+}$ store, the sacroplasmic reticulum. A schematic representation of electrically coupled SMCs is shown in Fig. 1. The elevation of the intracellular $\text {Ca}^{2+}$ concentration causes the membrane potential to increase rapidly, hence the cell membrane is depolarised, and this results in the opening of the $\text {K}^{+}$ channels. The efflux of $\text {K}^{+}$ then leads to the repolarisation of the cell. The $\text {Ca}^{2+}$ binds to the calmodulin in the cytoplasm to activate the myosin light chain kinase enzyme which results in myosin interaction with actin filaments to produce contractile activity. The repetition of this activity results in periodic oscillations that elicit vasomotion, that is, the contraction and relaxation of the vessel’s cell wall.

Oscillations can be driven by applied current (Hodgkin and Huxley 1952), agonists (Sneyd et al. 1995; Koenigsberger et al. 2005), temperature (Anatoly et al. 2013; Fillafer and Schneider 2013), and pressure (Kubanek et al. 2018). Several experimental studies have investigated the electrical activity induced by external stimuli in excitable cells (Friel 1995; Latchoumane et al. 2018; Liang et al. 2019); see also (Roth 1994; Farhy 2004; Koenigsberger et al. 2005; Izhikevich 2007; Fatoyinbo et al. 2022a, b)for computational studies.

Communication between cells, primarily excitable and non-excitable cells, helps regulate a wide range of cellular activities, for example, receiving and transmitting signals in the central nervous system (Duan et al. 2008; Keener and Sneyd 2009), the release of hormones into extracellular fluid in endocrine cells (Schwartz 2000; Nakanishi 2006; Combarnous and Nguyen 2020), and contractile activity in muscles (Mége et al. 1994; Matchkov 2010; Tirziu et al. 2010; Bian et al. 2015). SMCs connected to their immediate neighbors through different mechanisms (Jongsma and Wilders 2000; Giepmans 2004; Shimizu and Stopfer 2013) and coupled through gap junctions which can be one of three types: $\text {Ca}^{2+}$, inositol trisphosphate ($\text {IP}_{3}$), or membrane potential (electrical) (Koenigsberger et al. 2004; Haddock and Hill 2005; Koenigsberger et al. 2005). Gap junctional communications have been observed in other cell types, including germ cells in testis (Decrouy et al. 2004), fibroblasts (Azzam et al. 2001), and astrocytes (Giaume and McCarthy 1996).

The dynamics across a large number of coupled cells can form simple travelling waves, or complex spatiotemporal patterns. For example, as revealed in experiments, spiral waves during heart contractions can cause cardiac arrhythmia (Hwang et al. 2005; Pandit and Jalife 2013). Epileptic seizures in the cortex and hallucinations in the retina or visual cortex can be induced by travelling waves (Traub et al. 1993; Huang et al. 2004; Pinto et al. 2020; Pearce 2015).

When the number of cells is large, such dynamics can be well modelled by reaction-diffusion equations, as done originally by Turing (1952). In this framework each cell is described by ordinary differential equations that are usually strongly related to those given by Hodgkin and Huxley (1952), while communication between cells is captured by a diffusion term.

Spatiotemporal patterns can arise via diffusion-driven instability (Turing patterns) or other means. In ecology, the Lotka-Volterra model for two interacting species exhibits both Turing and non-Turing patterns when a diffusion term is added (Banerjee and Banerjee 2012; Shi and Ruan 2015; Liu et al. 2020). In epidemiology, spatial patterns have been observed in diffusive epidemic models designed to investigate the spread and control of infectious diseases (Jia et al. 2018; Chang et al. 2020). Also, various patterns have been observed in cellular dynamics (Izhikevich 2007; Ramos 2002; Kaper and Vo 2018; Vo et al. 2020) and physical and mechanical systems (Paul et al. 2003; Perez-Londoño et al. 2010; Hens et al. 2015).

There are many studies of spatiotemporal patterns in systems of excitable cells (Fujii and Tsuda 2004; Hartle and Wackerbauer 2017; Keplinger and Wackerbauer 2014; Lafranceschina and Wackerbauer 2014; Mondal et al. 2018; Calim et al. 2018). These have involved the Fitzhugh-Nagumo equations (Tsyganov et al. 2014), the Morris–Lecar equations (Meier et al. 2015; Mondal et al. 2019), and the Wilson-Cowan equations (Ali et al. 2016), for example. These studies focused on patterns and waves that are driven by applied current. The purpose of this paper is to stress that applied current is not necessary for spatiotemporal patterns to occur.

Such *pacemaker dynamics* have been identified experimentally in the gastrointestinal tract, urinary tract, lymphatic vessels, arteries, and veins Tomita (1981); Hashitani et al. (1996); Fukuta et al. (2002); Van Helden (1993); McHale et al. (2006). There have been several computational studies of pacemaker dynamics in SMCs (Youm et al. 2006; Rihana et al. 2009; Cho et al. 2012; Ho et al. 2016). Youm et al. (2006) modelled the pacemaker activity of interstitial cells of Cajal in the gastrointestinal tract. They found that spontaneous electrical activity is triggered by efflux of $\mathrm{Ca}^{2+}$ from the sacroplasmic recticulum mediated by $\mathrm{IP}_{3}$. In the work of Rihana et al. (2009) spontaneous electrical activity and contraction in single uterine SMCs during the gestation period is explored. Different ionic channels involved in uterine excitability at term are identified, the model reproduces results observed *in vivo*. Also pacemaker dynamics has been described in chemical and ecological models (Merkin and Sadiq 1996; Merkin et al. 1996; Or-Guil et al. 2001; Pal et al. 2019)

In this paper we focus on pacemaker electro-mechanical coupling activity in arterial SMCs due to changes in the vessel’s transmural pressure, that is, the pressure gradient across the vessel wall. We study a spatially extended two-variable nondimensionalised Morris–Lecar model with no applied current. Without diffusion the model is a reduced form of the three-dimensional ODE model of (Gonzalez-Fernandez and Ermentrout 1994) for vasomotion in SMCs of small arteries. Our previous work (Fatoyinbo et al. 2020) showed how, without diffusion, oscillations arise via Type I and Type II excitability. This distinction between the two types of excitability was first described by Hodgkin (1948). For Type I excitability oscillations arise via a saddle-node on invariant circle (SNIC) bifurcation, whereas for Type II excitability oscillations arise via a Hopf bifurcation (Rinzel and Ermentrout 1999).

In this paper we show how diffusion induces spatiotemporal patterns as well as travelling fronts and pulses. We start in Sect. 2 by stating the model equations. Then in Sect. 3 we summarise the dynamics of the model without diffusion using the voltage associated with the $\mathrm{K}^{+}$ and $\mathrm{Ca}^{2+}$ channels as bifurcation parameters. In Sect. 4 we show that the spatiotemporal patterns that emerge are non-Turing patterns due to violation of Turing’s instability criteria. Numerical simulations of the reaction-diffusion model are carried out in Sect. 5. Various spatiotemporal patterns including travelling pulses and fronts are explored. The existence of the travelling waves is analysed in Sect. 6. Finally conclusions are presented in Sect. 7.

## A Nondimensionalised Morris–Lecar System with Diffusion

We consider a nondimensionalised reaction-diffusion system to model the dynamics of a population of coupled SMCs through passive electrical coupling of adjacent cells. The reaction term in the model is based on our previous study on an isolated SMC (Fatoyinbo et al. 2020). The model equations are

1 $$\begin{aligned} \frac{\partial V}{\partial \tau }&=D\frac{\partial ^{2} V}{\partial X^{2}}-\bar{g}_{L}(V-\bar{v}_{L})-\bar{g}_{K}N(V-\bar{v}_{K})-\bar{g}_{\text {Ca}}M_{\infty }(V)(V-1), \end{aligned}$$

2 $$\begin{aligned} \frac{\partial N}{\partial \tau }&=\lambda (V)(N_{\infty }(V)-N), \end{aligned}$$

where $V(X,\tau )$ is the membrane potential and $N(X,\tau )$ is the fraction of open $\text {K}^{+}$ channels. The system parameter $D \ge 0$ is the diffusion coefficient, $\bar{g}_\mathrm{L}$, $\bar{g}_\mathrm{K}$, and $\bar{g}_\mathrm{Ca}$ are conductances per unit area for the leak, potassium, and calcium currents, respectively, while $\bar{v}_\mathrm{L}$ and $\bar{v}_\mathrm{K}$ are the corresponding Nernst reversal potentials (equilibrium potentials). The fraction of open calcium [potassium] channels at steady state $M_{\infty }$ [$N_\infty$] and the time scale for the opening of the potassium channel, $\lambda (V)$ are:

3 $$\begin{aligned} M_{\infty }(V)&=\frac{1}{2}{}\left( 1+\tanh \left( \frac{V-\bar{v}_{1}}{\bar{v}_{2}}\right) \right) , \end{aligned}$$

4 $$\begin{aligned} N_{\infty }(V)&=\frac{1}{2}\left( 1+\tanh \left( \frac{V-\bar{v}_{3}}{\bar{v}_{4}}\right) \right) ,\end{aligned}$$

5 $$\begin{aligned} \lambda (V)&=\psi \cosh \left( \frac{V-\bar{v}_{3}}{2\bar{v}_{4}}\right) , \end{aligned}$$

where $\bar{v}_1$ and $\bar{v}_3$ measure the potential at which potassium and calcium channels are half-opened, $\psi$ is a time constant, and $\bar{v}_2$ and $\bar{v}_4$ are additional parameters. Unless otherwise specified, we use the following values of the parameters: $\bar{v}_{1}=-0.2813$, $\bar{v}_{2}=0.3125$, $\bar{v}_{3}=-0.1380$, $\bar{v}_{4}=0.1812$, $\psi =0.1665$, $\bar{v}_\mathrm{L}=-0.875$, $\bar{v}_\mathrm{K}=-1.125$, $\bar{g}_\mathrm{L}=0.25$, $\bar{g}_\mathrm{K}=1.0$, and $\bar{g}_\mathrm{Ca}=0.4997$.

In this paper, we consider a one-dimensional spatial domain $\Omega = [-L, L]$ for the values of *X*. At the boundaries, $X = \pm L$, we use no-flux boundary conditions. However we are primarily concerned with the dynamics that emerges away from the boundaries. Further, the diffusion coefficient *D* can be scaled to any value by scaling the spatial variable *X* appropriately. Thus the value of *D* only affects the speed at which dynamics propagates, not the types of dynamics that arise. For these reasons, the values of *D* and *L* will not be important to the spatiotemporal patterns that we describe below.

**Fig. 2:**
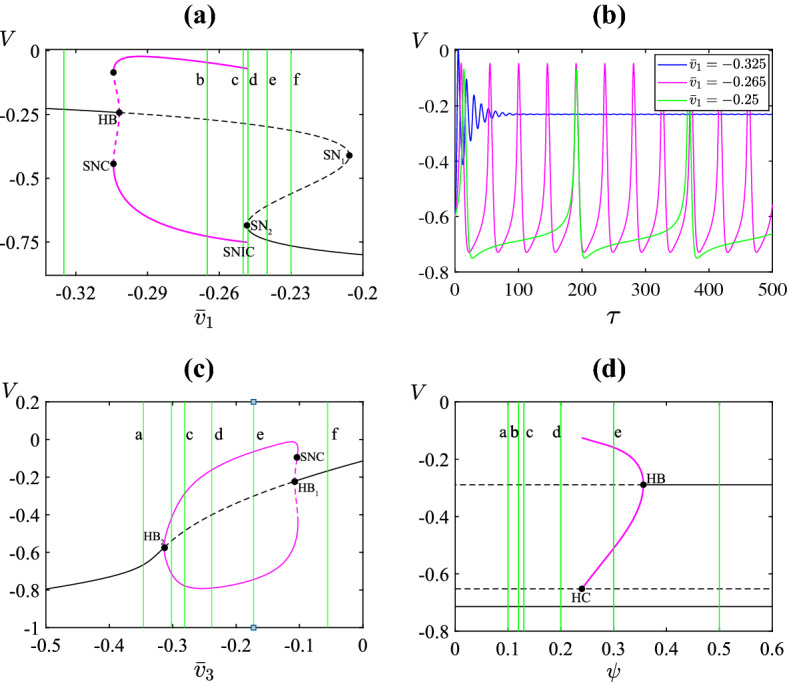
Panels **a** and **b** show a bifurcation diagram and the time series of (1)–(2) with $D=0$ using $\bar{v}_1$ as the bifurcation parameter. Panels **c** and **d** show the bifurcation diagrams with $\bar{v}_3$ and $\psi$ as the bifurcation parameters. In each bifurcation diagram all parameters (except the one being varied) are fixed at the values listed in Sect. 2. Black [magenta] curves correspond to equilibria [limit cycles]. Solid [dashed] curves correspond to stable [unstable] solutions. The vertical lines indicate the parameter values used in Figs. 3, 7, and 8. HB: Hopf bifurcation; SN: saddle-node bifurcation (of an equilibrium); SNC: saddle-node bifurcation of a limit cycle; SNIC: saddle-node on an invariant circle bifurcation; HC: homoclinic bifurcation (Color figure online)

## The Dynamics of a Single Cell

In this section we summarise some of the results of Fatoyinbo et al. (2020) for the dynamics of (1)–(2) in the absence of diffusion, i.e. $D=0$. We show how stable oscillations are created through either Type I or Type II excitability and consider the effect of varying $\psi$ (not done in Fatoyinbo et al. (2020)). This is important to the nature of the spatiotemporal dynamics described in Sect. 5. More details on the dynamics of a single cell can also be found in (Fatoyinbo 2020).

Figure 2 shows bifurcation diagrams as $\bar{v}_{1}$, $\bar{v}_{3}$, and $\psi$ are varied from their values as listed in Sect. 2. These were computed numerically using auto (Doedel et al. 2012). Spontaneous oscillations in (1)–(2) are triggered by a change in transmural pressure, and therefore, we use pressure-dependent parameters, $\bar{v}_{1}$ and $\bar{v}_{3}$, as bifurcation parameters.

Figure 2 a shows the result of varying $\bar{v}_1$. The system has a unique equilibrium except between saddle-node bifurcations $\mathrm{SN}_1$ and $\mathrm{SN}_2$ where there are three equilibria: one stable (lower branch) and two unstable (middle and upper branch). As the value of $\bar{v}_1$ is increased from the smallest value shown in the diagram, the upper equilibrium branch loses stability in a subcritical Hopf bifurcation (HB). The unstable limit cycle produced here gains stability via a saddle-node bifurcation (SNC). Upon further increasing the value of $\bar{v}_{1}$, the stable limit cycle is destroyed at the saddle-node bifurcation $\mathrm{SN}_2$. This is an example of a saddle-nodle on invariant circle bifurcation (SNIC) where the limit cycle is replaced by a heteroclinic connection between the two equilibria (Kuznetsov 1995). As a consequence, the period of the limit cycle approaches infinity as the bifurcation is approached. Here the system displays Type I excitability as stable oscillations are created in a SNIC bifurcation by appropriately decreasing the value of $\bar{v}_1$. Figure 2b shows the temporal dynamics of the membrane potential *V* for $\bar{v}_{1}=-0.325, -0.265$ and $-0.25$ in Fig. 2a.

Next we vary the value of $\bar{v}_3$. As shown in Fig. 2c, as we increase the value of $\bar{v}_3$ a unique equilibrium loses stability in a supercritical Hopf bifurcation $\mathrm {HB}_1$ then regains stability in a subcritical Hopf bifurcation $\mathrm {HB}_2$. The stable oscillations are created at $\mathrm {HB}_1$ with finite period. They subsequently lose stability at a saddle-node bifurcation and terminate at $\mathrm {HB}_2$. In this case the system displays Type II excitability since the periodic oscillations arises through a Hopf bifurcation.

Finally Fig. 2d shows how the dynamics changes under variation to the value of $\psi$. The system has three equilibria for all values of $\psi >0$. For relatively low and intermediate values of $\psi$, there exist one stable (lower branch) and two unstable (upper and middle branch) equilibria. By increasing $\psi$, a stable limit cycle emanates through a homoclinic bifurcation (HC) and upon further increase of $\psi$ terminates in a supercritical Hopf bifurcation (HB). As in Fig. 2c the excitability here is Type I. Between the homoclinic and Hopf bifurcations the system is bistable as the limit cycle coexists with a stable equilibrium. As shown in Fatoyinbo et al. (2020), for different parameter values the system has three coexisting stable solutions.

## Linear Stability Analysis

Alan Turing (1952) hypothesised that spatially inhomogeneous patterns may arise in a reaction-diffusion system if a spatially homogeneous steady state is stable in the absence of diffusion and destabilised as result of diffusion. Such instability is referred to as diffusion-driven instability or Turing instability. The conditions required for the onset of Turing instability have been well studied (Alonso et al. 2002; Shoji et al. 2003; Murray 2003; Banerjee and Banerjee 2012; Krause et al. 2021). Here we perform a linear stability analysis of (1)–(2) around a spatially homogeneous steady state and show that the conditions for Turing instability are not satisfied for this system.

As shown in the previous section, in the absence of diffusion (1)–(2) typically has one or three equilibria and the stability of these depends on the values of the parameters. Here let $(V^*,N^*)$ be a stable equilibrium of (1)–(2) with $D=0$ (i.e. no diffusion) for some combination of parameter values. Then for (1)–(2) with $D > 0$, $(V^*, N^*)$ represents a spatially homogeneous state.

Let $\big (V_{0}(X,\tau ),N_{0}(X,\tau )\big )$ represent the perturbation of a solution to (1)–(2) from the steady state, i.e.

6 $$\begin{aligned} \begin{pmatrix} V_{0}\\ N_{0} \end{pmatrix}=\begin{pmatrix} V- V^{*}\\ N-N^{*} \end{pmatrix}. \end{aligned}$$

By linearising (1)–(2) about $(V^*,N^*)$, we obtain the following leading-order approximation to the dynamics of the perturbation:

7 $$\begin{aligned} \begin{pmatrix} V_{0}\\ N_{0} \end{pmatrix}_{\tau }=\begin{pmatrix} D &{} 0\\ 0 &{} 0 \end{pmatrix}\begin{pmatrix} V_{0}\\ N_{0} \end{pmatrix}_{XX}+\begin{pmatrix} f_{V} &{} f_{N}\\ g_{V} &{} g_{N} \end{pmatrix}\begin{pmatrix} V_{0}\\ N_{0} \end{pmatrix}. \end{aligned}$$

The second matrix in (7) is the Jacobian matrix of (1)–(2) evaluated at $(V^*,N^*)$. By directly differentiating (2) with respect to *N*, we obtain

8 $$\begin{aligned} g_{N}&=-\psi \cosh \left( \frac{V^*-\bar{v}_{3}}{2\bar{v}_{4}}\right) . \end{aligned}$$

Formulas for the other three entries in the Jacobian matrix will not be needed.

We now look for a solution to (7) of the form $(V_{0}, N_{0})(X,\tau )=\varvec{\beta } e^{(\lambda \tau +ikX)}$, where $\varvec{\beta }$ is a constant vector, $\lambda$ is the growth rate of perturbation in time, and *k* is the wave number. While there are many such solutions, we will show that all must have $\lambda <0$. This implies that for any sufficiently small perturbation (6), the corresponding solution to (1)–(2) decays to $(V^*,N^*)$ as $t\rightarrow \infty$, hence the steady state is not destabilised (Murray 2003). By substituting the given form into (7),

9 $$\begin{aligned} \begin{pmatrix} -k^2D+f_{V}-\lambda &{} f_{N}\\ g_{V} &{} g_{N}-\lambda \end{pmatrix} \varvec{\beta }=\begin{pmatrix} 0 \\ 0 \end{pmatrix}. \end{aligned}$$

Equation (9) is homogeneous in $\varvec{\beta }$, thus has a nontrivial solution only if the matrix in (9) is singular.

This implies

10 $$\begin{aligned} \lambda =\frac{T}{2}\pm \frac{\sqrt{T^{2}-4\Delta }}{2}, \end{aligned}$$

where $T=-k^2D+f_{V}+g_{N}$ and $\Delta =-k^2Dg_{N}+f_{V}g_{N}-g_{V}f_{N}$ denote the trace and determinant of the matrix in (9) when $\lambda = 0$. By assumption $(V^*,N^*)$ is stable in the absence of diffusion, therefore

11 $$\begin{aligned} f_{V}+g_{N}<0, \qquad f_{V}g_{N}-f_{N}g_{V}>0. \end{aligned}$$

But from (8) we always have $g_{N}<0$ because $\psi >0$ for physical reasons. Therefore $T<0$ and $\Delta >0$, thus $\lambda <0$ for any $D>0$. Thus $(V^*,N^*)$ is not destabilised by the inclusion of diffusion and so the spatiotemporal patterns that we describe below are not due to Turing instability. Similarly, Klika et al. (2012) provided conditions for the emergence of patterns in reaction-diffusion systems outside the classical Turing mechanism. They found that for arbitrarily large values of wave number $k^2$ the system is destabilised. Also, in line with our results, it has been reported from previous studies on coupled PDE-ODEs models that patterns cannot occur unless the system is unstable in the absence of diffusion (Härting and Marciniak-Czochra 2014; Marciniak-Czochra et al. 2017).

## Spatiotemporal Dynamics of the Full Model

In this section we explore the effect of varying $\bar{v}_{1}$, $\bar{v}_{3}$ and $\psi$ on the spatiotemporal dynamics of the reaction-diffusion system (1)–(2). Since the patterns are not due to Turing instability, as shown in Sect. 4, we will investigate spatiotemporal dynamics for a wide range of parameter values, in particular where the steady states may be stable or unstable. We show numerically that a wide range of spatiotemporal patterns can occur, including travelling pulses, travelling fronts, and spatiotemporal chaos.

The system (1)–(2) was solved numerically by using the method of lines. We used a second-order central finite difference approximation to the spatial derivative using 1000 *X*-values per unit interval, and a standard numerical scheme for the time derivative (ode15s in matlab) (Schiesser and Griffiths 2009; Hiptmair et al. 2010). All numerical simulations use no-flux boundary conditions for $X\in [-L,L]$ and initial conditions

12 $$\begin{aligned} V(0,X)=V^*+G(X) \text {and} N(0,X)=N^*, \end{aligned}$$

where $(V^*,N^*)$ is a homogeneous steady state of (1)–(2). Different functions *G*(*X*) (specified below) provide different perturbations from the steady state. As mentioned in Sect. 2, a linear coordinate change can be applied to (1)–(2) to scale the value of $D>0$ to any positive number; in all simulations below, we use $D=0.0001$.

### The Effect of the Parameters $\bar{v}_{1}$, $\bar{v}_{3}$, and $\psi$

Now we examine the spatiotemporal patterns exhibited by (1)–(2) for the values of $\bar{v}_{1}$, $\bar{v}_{3}$, and $\psi$ marked a-f in Fig. 2. In this initial condition (12), we use the Gaussian perturbation,

13 $$\begin{aligned} G(X)=A_0 \,\mathrm{exp} \left( \frac{-X^2}{2 \sigma ^2} \right) , \end{aligned}$$

with $$A_{0}=0.3$$ and $$\sigma =0.1$$.

**Fig. 3:**
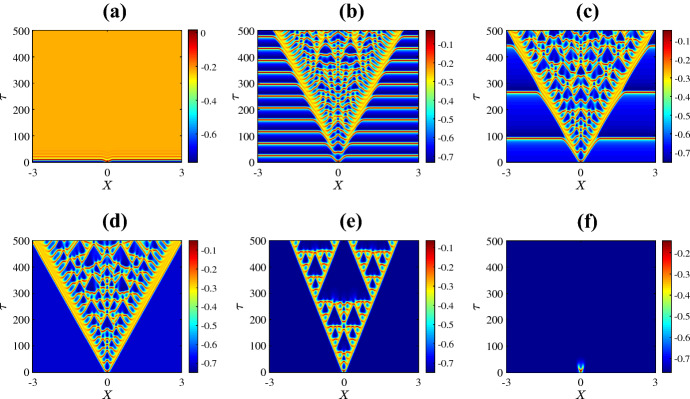
Space-time plots of the membrane potential *V* for the values of $\bar{v}_{1}$ marked in Fig. 2a. Specifically **a** $-0.325$; **b** $-0.265$; **c** $-0.25$; **d** $-0.248$; **e** $-0.240$; and **f** $-0.230$. The initial condition is (12) with (13), using the upper equilibrium branch of Fig. 2a for the steady state $(V^*,N^*)$, and all other parameters are fixed as in Sect. 2 (Color figure online)

Figure 3 shows the resulting spatiotemporal patterns for different values of $\bar{v}_{1}$. For low values of $\bar{v}_{1}$ the system has a unique homogeneous steady state (the upper equilibrium branch in Fig. 2a). This steady state is stable and the solution quickly converges to the steady state as in Fig. 3a. Instead with $\bar{v}_1$ just to the right of the Hopf bifurcation, a complex spatiotemporal pattern emerges, as shown in Fig. 3b. The solution starts as a pulse at the centre of the domain due to the initial perturbation. Then the pulse splits into two propagating pulses that transition to time-periodic oscillations with inhomogeneous patterns at the back as they move across the domain. Outside the patterned region the solution is periodic corresponding to the limit cycle of the system with no diffusion. Similar behaviour is observed for values of $\bar{v}_{1}$ between the Hopf bifurcation and the SNIC bifurcation. For example in Fig. 3c we have used $\bar{v}_1 = 0.25$. This is very close to the SNIC bifurcation so now the oscillations outside the patterned region are of particularly high period. Such patterns are sometimes termed *generalised travelling waves* (Vakulenko and Volpert 2001)

**Fig. 4:**
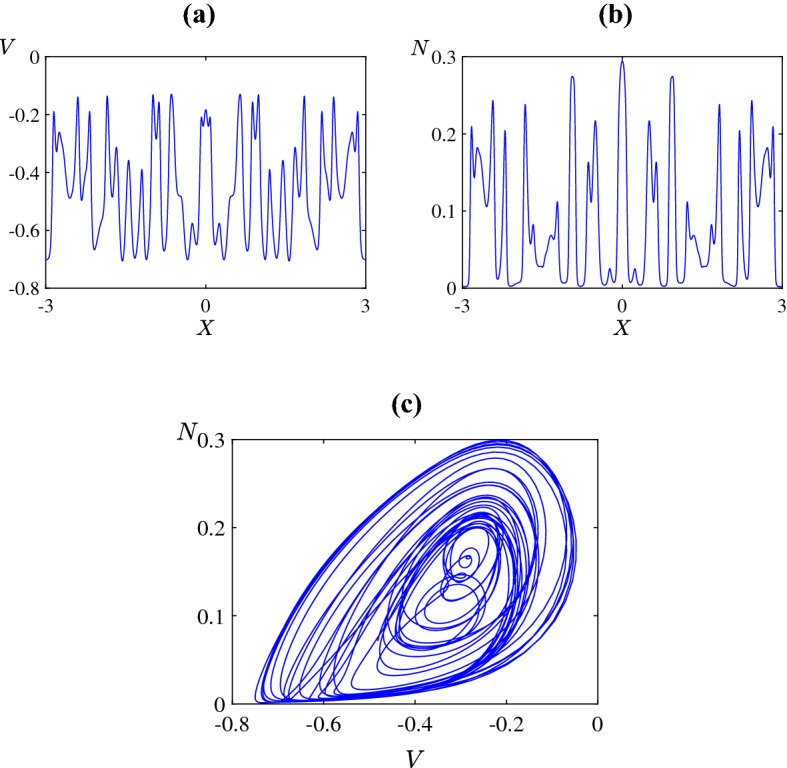
The spatial distribution of the **a** membrane potential *V*; **b** fraction of open potassium channels *N* with $\bar{v}_1=-0.25$ (as in Fig. 3c) and $\tau =500$. Panel **c** shows the variables plotted against each other over all $-3< X<3$ (Color figure online)

Beyond the SNIC bifurcation, as in Fig. 3d, we again observe complex spatiotemporal patterns but now oscillations do not occur outside the patterned region because the system with no diffusion no longer has a stable limit cycle. With a yet larger value of $\bar{v}_1$ the pattern forms a relatively ordered triangular structure bearing an interesting resemblance to the Sierpinski triangle. Numerical simulations performed over a longer time scale suggest that this structure persists indefinitely. Figure 4 shows a typical profile of the solution at a large time. By increasing the value of $\bar{v}_1$ further, as in Fig. 3f, patterns are no longer observed. Here the solution simply decays to the stable homogeneous steady state (the lower equilibrium branch of Fig. 2a).

**Fig. 5:**
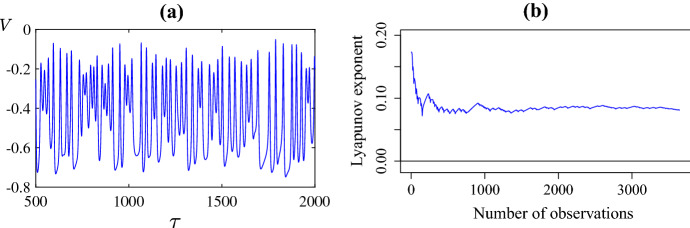
**a** A time series of the membrane potential *V* at spatial point $X=-1$ for the parameter values of Fig. 3c; **b** A plot of the Lyapunov exponent as a function of the number of observations (data points) produced by the numerical package **DChaos** of R (Color figure online)

**Fig. 6:**
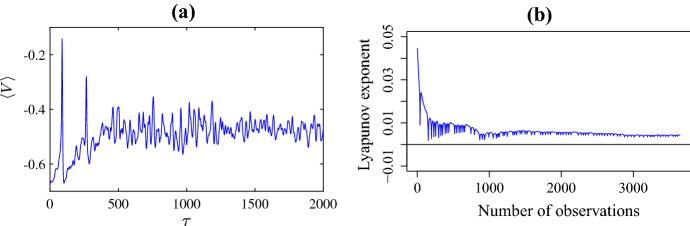
**a** A plot of the spatial average of the membrane potential *V* against time for the pattern shown in Fig. 3c; **b** A plot of the Lyapunov exponent as a function of the number of observations (data points) produced by the numerical package **DChaos** of R (Color figure online)

The irregular nature of the patterns in Fig. 3 strongly suggests that the dynamics is chaotic. To obtain further evidence of this we numerically estimated the maximal Lyapunov exponent through the **DChaos** package of the software R (Sandubete and Escot 2021). Specifically we considered the parameter values of Fig. 3c at the fixed spatial value $X=-1$ (other values gave similar results) and applied the numerical algorithm to the resulting time series with transient dynamics removed (shown in Fig. 5a). This produced an estimate for the Lyapunov exponent as $\lambda = 0.12$, with greater than $95\%$ confidence that the true value is greater than zero and the solution is chaotic. Figure 5b illustrates the convergence of the algorithm to its final estimate over the number of data points used. Further Fig. 6a shows the spatial average as a function of time. This also shows irregular oscillations and here **DChaos** estimated the maximal Lyapunov exponent as $\lambda = 0.04$ again with greater than $95\%$ confidence that the true value is positive further reinforcing our claim that the dynamics is indeed chaotic.

**Fig. 7:**
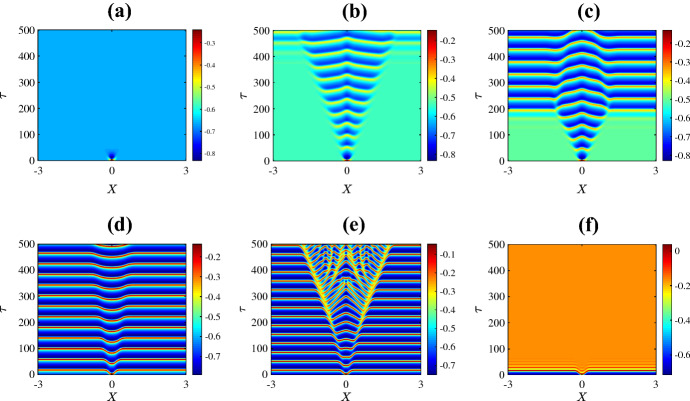
Space-time plots of the membrane potential *V* for the values of $\bar{v}_{3}$ marked in Fig. 2c. Specifically **a** $-0.3462$; **b** $-0.3019$; **c** $-0.2813$; **d** $-0.2384$; **e** $-0.1725$; and **f** $-0.05565$. The initial condition is (12) with (13), using the stable and unstable equilibrium branch of Fig. 2c for the steady state $(V^*,N^*)$, and all other parameters are fixed as in Sect. 2 (Color figure online)

Now we study the spatiotemporal behaviour of the model by varying $\bar{v}_{3}$ and keeping all other parameters fixed as in Sect. 2. Recall that in this case the system in the absence of diffusion exhibits supercritical and subcritical Hopf bifurcations (see Fig. 2c). The results of numerical simulations are shown in Fig. 7. For extremely low values of $\bar{v}_{3}$, the system returns quickly to the homogeneous steady state. Between the Hopf bifurcations, where the system in the absence of diffusion has a stable limit cycle, we observe mostly homogeneous oscillations corresponding to this limit cycle (see Fig. 7b–e). In panels (b) and (c) away from $X=0$ where the perturbation is applied, it takes some time for the solution to settle to oscillatory behaviour because the initial condition is set very near the value of the unstable steady state. In panels (d) and (e) oscillations develop across the domain relatively quickly. In panel (e), which is just before the subcritical Hopf bifurcation, the initial stimulus creates a pulse of propagating action potentials. For values of $\bar{v}_3$ beyond the subcritical Hopf bifurcation and subsequent saddle-node bifurcation SNC (see Fig. 2c), periodic oscillations can be observed for a short time across the entire domain, then stabilise to the homogeneous steady state, as in Fig. 7f.

**Fig. 8:**
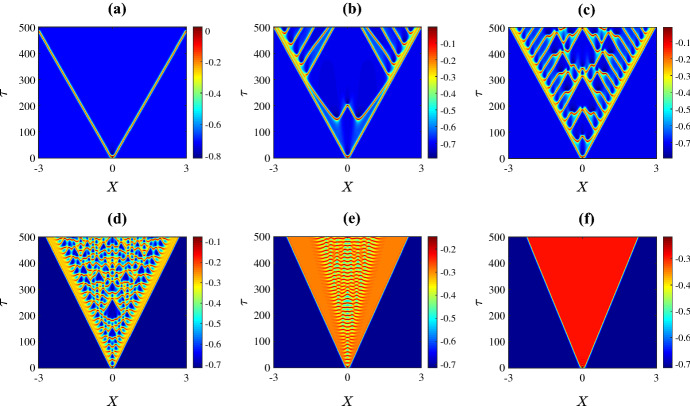
Space-time plots of the membrane potential *V* for values of $\psi$ as marked in Fig. 2d. Specifically **a** 0.1; **b** 0.12; **c** 0.13; **d** 0.2; **e** 0.3; and **f** 0.5. The initial condition is (12) with (13), using the upper equilibrium branch of Fig. 2a for the steady state $(V^*,N^*)$, and all other parameters are fixed as in Sect. 2. The solution transitions from propagating pulses travelling in opposite direction to complex spatiotemporal patterns to fronts travelling in opposite direction (Color figure online)

Finally Fig. 8 shows spatiotemporal patterns for the various values of $\psi$ marked in Fig. 2d. For extremely low values of $\psi$, the initial perturbation creates a pulse at the centre of the domain and as time progresses the pulse splits into two travelling pulses propagating in opposite directions at the same speed (Fig. 8a). A slight increase in the value of $\psi$ leads to a destabilisation of the pulses that results in an initiation of secondary pulses travelling in the opposite direction to the primary pulses (Fig. 8b). By increasing the value of $\psi$ further, we are able to see within the $\tau = 500$ time frame that the secondary pulses collide with one another and eventually irregular oscillations disseminate across the spatial domain (Fig. 8c–d). Interestingly, as $\psi$ is varied past the homoclinic bifurcation, the unstable pulses transition to travelling fronts connecting a stable steady state to an unstable state with irregular oscillations at the back of the fronts (Fig. 8e). As the value of $\psi$ is increased further, the upper equilibrium branch gains stability at the Hopf bifurcation so beyond this bifurcation the system has two stable steady states. In this case the fronts connect one stable steady state to the other (Fig. 8f).

**Fig. 9:**
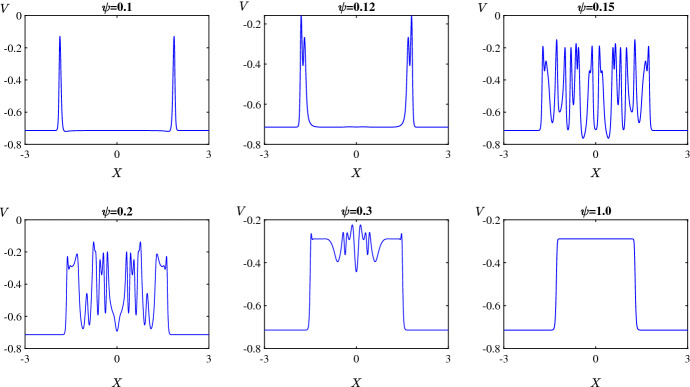
Solution profiles at time $\tau =300$ showing the transitions from travelling pulses to spatiotemporal chaos and to fronts (Color figure online)

Figure 9 shows the solution at $\tau = 300$ for the six values of $\psi$ used in Fig. 8. This shows how increasing the value of $\psi$ causes the two travelling pulses to transition into two travelling fronts via an intermediate phase of spatiotemporal chaos.

**Fig. 10:**
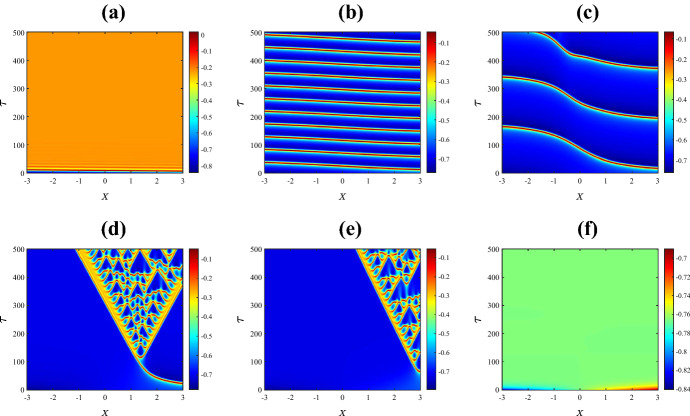
Space-time plots using the same parameter values as Fig. 3 but now with the perturbation function (14) in the initial condition (12) (Color figure online)

### Numerical Simulations with Alternate Initial Conditions

**Fig. 11:**
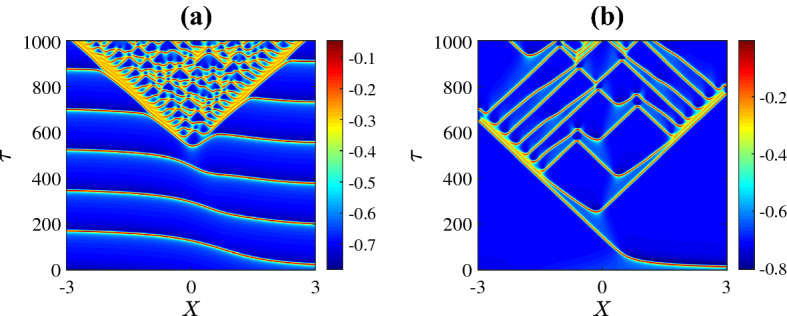
Space-time plots of the membrane potential *V* using a longer time frame than other plots. The parameter values and initial conditions in panel **a** are the same as Fig. 10f, and in panel **b** are same as Fig. 8b (Color figure online)

In this section we consider other perturbation functions *G*(*X*) in the initial condition (12) to investigate how the initial condition affects the patterns that develop. First we consider

14 $$\begin{aligned} G(X)=\epsilon X, \end{aligned}$$

with $\epsilon =0.025$. Figure 10 shows the resulting spatiotemporal patterns for different values of $\bar{v}_1$. Specifically the six plots use the same parameter values as the corresponding plots in Fig. 3. In panels (a) and (f) of Fig. 10 the solution simply settles to the stable equilibrium of the system in the absence of diffusion (as in Fig. 3). In panels (b) and (c) the initial condition is insufficient to generate the spatiotemporal chaos that was observed in Fig. 3 within the $\tau =500$ time frame. By simulating for a longer time we found that in (b) the solution appeared to converge to homogeneous oscillations matching the stable limit cycle of the system in the absence of diffusion, while in (c) spatiotemporal chaos did arise shortly after $\tau = 500$, and this is shown in Fig. 11a. Finally in panels (d) and (e) we do observe spatiotemporal chaos. The particular patterns that emerge appear to have the same features as those in Fig. 3 suggesting that for both initial conditions the solution is converging to the same attractor.

For other values of the parameters and other initial conditions we similarly observed that, broadly speaking, the dynamics of (1)–(2) settled to the same long-time behaviour as that described in Sect. 5.1. For example using the parameter values of Fig. 8b, when the initial condition is changed from (13) to (14) the result is Fig. 11b which evidently exhibits a similar structure. We conclude that the profile of the initial perturbation does not seem to change the types of spatiotemporal patterns that are produced by the model.

## Travelling Wave Analysis

**Fig. 12:**
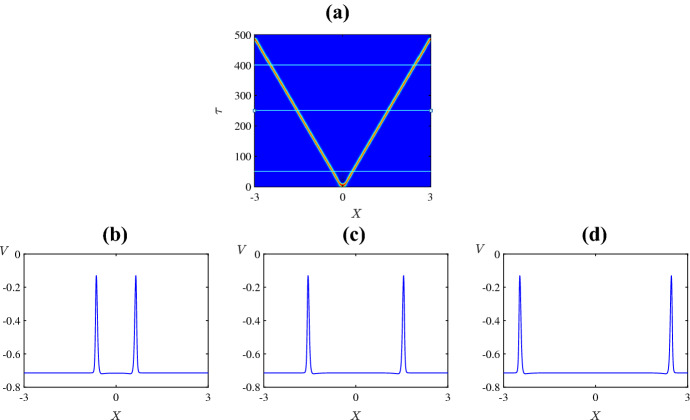
A reproduction of Fig. 8a and plots of the solution profile at the values of $\tau$ that are marked by horizontal lines (Color figure online)

**Fig. 13:**
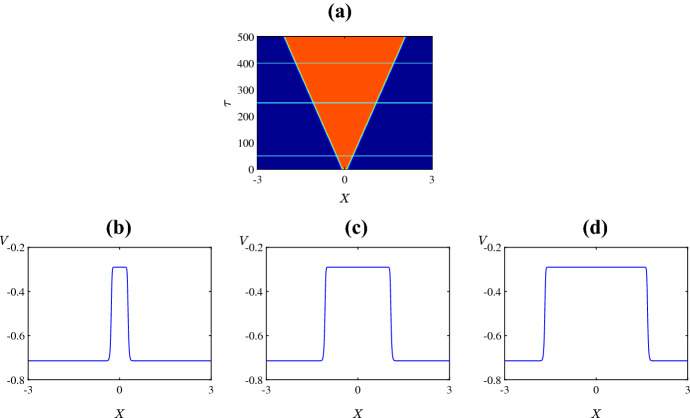
A reproduction of Fig. 8f and plots of the solution profile at the values of $\tau$ that are marked by horizontal lines (Color figure online)

For the travelling waves analysis, we will focus on the values of $\psi$ where the numerical simulations of (1)–(2) result in travelling pulses and fronts, respectively. For example, when $\psi =0.1$ two stable counter-propagating pulses are created, and they travel across the domain at approximate speed $c=0.006182$ (see Fig. 8a). Figure 12a shows the pulses and Fig. 12b–d are solution profiles at times $\tau =50$, 250, 400. Also, when $\psi =0.5$ two stable counter-propagating fronts are created, and they travel across the domain at speed $c=0.004155$ (see Fig. 8f). The fronts are shown in Fig. 13a, b–d are solution profiles at the same three times. The given wave speeds have been estimated directly from the numerical simulation results.

In the coming section, we introduce the travelling wave variable to transform (1)–(2) to a set of three ODEs and approximate the travelling wave solutions numerically. This allows us to find the homoclinic and heteroclinic trajectories that correspond to the travelling pulse and front solutions, respectively.

### Existence of Travelling Waves

To describe the travelling wave profile we consider travelling waves with unknown wave speed $c>0$. By introducing the travelling wave variable, $\zeta = X-c\tau$, the model (1)–(2) becomes

15 $$\begin{aligned} \begin{pmatrix} V\\ N \end{pmatrix}_{\tau }=D\begin{pmatrix}V\\ 0 \end{pmatrix}_{\zeta \zeta }+\begin{pmatrix}cV\\ cN\end{pmatrix}_{\zeta }+\begin{pmatrix}f(V,N)\\ g(V,N)\end{pmatrix}, \end{aligned}$$

where

$$\begin{aligned} f(V,N)&=-\bar{g}_{L}(V-\bar{v}_{L})-\bar{g}_{K}N(V-\bar{v}_{K})-\bar{g}_{\text {Ca}}M_{\infty }(V)(V-\bar{v}_{\text {Ca}}),\\ g(V,N)&=\lambda _N(V)\big (N_{\infty }(V)-N\big ). \end{aligned}$$

Travelling waves are stationary solutions to (15) and satisfy

16 $$\begin{aligned} D\begin{pmatrix}V\\ 0 \end{pmatrix}_{\zeta \zeta }+c\begin{pmatrix}V\\ N\end{pmatrix}_{\zeta }+\begin{pmatrix}f(V,N)\\ g(V,N)\end{pmatrix}=0. \end{aligned}$$

We rewrite (16) as system of first order ODEs with $':=\frac{d}{d\zeta }$ by introducing a new variable $W=V'$ to obtain

17 $$\begin{aligned} \begin{aligned} V'&=W,\\ W'&=-\frac{1}{D}(cW+f(V,N)),\\ N'&=-\frac{1}{c}g(V,N). \end{aligned} \end{aligned}$$

The boundary conditions are

18 $$\begin{aligned} \lim _{\zeta \rightarrow +\infty }(V,W,N)(\zeta )=(V_{+},0,N_{+}), \lim _{\zeta \rightarrow -\infty }(V,W,N)(\zeta )=(V_{-},0,N_{-}), \end{aligned}$$

where $(V_{\pm },N_{\pm })$ are equilibria of (1)–(2). For a pulse $(V_+,N_+)$ and $(V_-,N_-)$ are the same equilibrium; for a front they are different equilibria.

A number of mathematical methods have been established to show the existence of travelling wave solutions in reaction-diffusion systems. These involve singular perturbation theory (Merkin and Sadiq 1996; Cornwell and Jones 2018), variational techniques (Chen and Choi 2015), and factorisation (Achouri 2016). We use the shooting method (Ermentrout 2002) to identify travelling waves and approximate their wave speed. This was achieved by numerically computing solutions to the travelling wave ODEs (17) for initial points perturbed from an equilibrium in a direction tangent to either its stable manifold or unstable manifold. In either case, this direction is given by an eigenvector of the Jacobian matrix of (17) evaluated at the equilibrium, and a formula for this matrix is provided in Appendix A. We adjusted the value of *c* until the solution was approximately homoclinic (in the case of a pulse) or heteroclinic (in the case of a front).

**Fig. 14:**
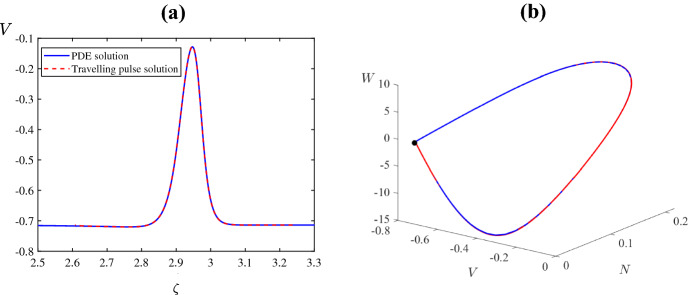
**a** The solution profile of (1)–(2) and a solution to the travelling wave ODEs (17) with $c=0.006116$, using the same parameter values as Fig. 8a **b** The same two solutions but plotted in the phase space of (17) (Color figure online)

We first consider the parameter values of Fig. 8a for which stable travelling pulses were observed. The equilibrium associated with these pulses is the lower-most equilibrium branch of Fig. 2c. For the travelling wave ODEs (17), this equilibrium has a one-dimensional unstable manifold. By performing the shooting method, we found that a solution approximating one branch of this manifold forms a homoclinic connection when $c = 0.006116$, approximately. This matches the speed of the pulses observed in Fig. 8a. A plot of the pulse profile for *V* is shown in Fig. 14a and its corresponding homoclinic trajectory in (*V*, *W*, *N*) phase space is shown in Fig. 14b. As expected the pulse profile extracted from our numerical solution to (1)–(2) matches the pulse solution obtained of the travelling wave ODEs (17).

**Fig. 15:**
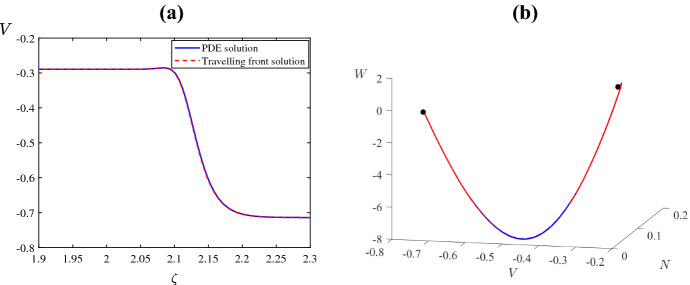
**a** The solution profile of (1)–(2) and a solution to the travelling wave ODEs (17) with $c=0.0043$ using the same parameter values as Fig. 8f **b** The same two solutions but plotted in the phase space of (17) (Color figure online)

Now we consider the parameter values of Fig. 8f for which our numerical solution produced two travelling fronts. These connect the lower-most and upper-most equilibrium branches of Fig. 2c. As equilibria of (17), these have one-dimensional stable manifolds. Consequently, we solved (17) backwards in time from an initial point near the upper equilibrium and adjusted the value of *c* until observing an approximately heteroclinic orbit. This produced $c = 0.0043$, approximately, matching the wave speed observed in Fig. 8f. The plot of the front profile for $V(\zeta )$ is shown in Fig. 15a, and its corresponding heteroclinic trajectory in (*V*, *W*, *N*) phase space is shown in Fig. 15b. The front obtained by the solution to (1)–(2) is also shown and seen to closely match the front profile of (17).

## Discussion

In this paper we used a reaction-diffusion model to investigate the collective dynamics of SMCs with passive electrical coupling. The main feature of the model is that it exhibits excitatory waves in response to mechanical stimulation even when the applied current is absent. In order to reveal the mechanisms that underpin these spatiotemporal dynamics, we have studied the model with simply one spatial dimension.

First we summarised and extended the bifurcation analysis of the system in the absence of diffusion given in Fatoyinbo et al. (2020). In particular the analysis reveals that the model can generate oscillations through Type I and Type II excitability.

This analysis is the starting point for explaining many aspects of the spatiotemporal dynamics of the full model, as detailed in Sect. 5. By using travelling wave coordinates, we were able to understand the occurrence of travelling pulses and fronts, Sect. 6. For instance, a stable travelling pulse transitions to a stable travelling front as the rate constant for the $\mathrm{K}^+$ channel is increased. In the absence of stable travelling pulses or fronts we observed what appears to be spatiotemporal chaos. We estimated the maximal Lyapunov exponent to show that the irregular behaviour is indeed chaotic. This chaos is caused by the presence of diffusion (because without diffusion the model consists of two ODEs). Overall we found that the long-term dynamics was independent of the choice of initial perturbation used in (12). We also showed in Sect. 4 that the spatiotemporal dynamics are not due to Turing instability.

The analysis in our paper demonstrates a biologically plausible system wherein complex spatiotemporal patterns can emerge from a system of SMCs. Moreover, these patterns are not driven by spatial inhomgeneities in the system, nor by Turing instability. We have observed patterns of excitation waves similar to those found in models of cardiac arrhythmia (Davidenko et al. 1992; Ermentrout and Rinzel 1996; Dodson and Sandstede 2019). The results in this study could be useful in improving our understanding of physiological responses and disorders in smooth muscle cells.

Several aspects of the spatiotemporal chaos remain to be explained. It would be useful to obtain a theoretical understanding for the speed at which the boundary of the chaotic region propagates. It would also be helpful to pinpoint bifurcations at which travelling pulses and fronts lose stability and these are likely to represent the onset of the chaotic dynamics. It would also be interesting to see what patterns occur when two or three spatial dimensions are considered.

## Data Availability

The matlab code we have used to produce the numerical simulations in this paper is available at https://github.com/hamfat

## Appendix A: The linearisation of the travelling wave ODE

Here we evaluate the Jacobian matrix of the travelling wave ODEs (17) at an arbitrary equilibrium $(V_{\pm },W_{\pm },N_{\pm })$. The Jacobian matrix is

19 $$\begin{aligned} J=\begin{pmatrix}0&{}1&{}0\\ -\frac{1}{D}f_{V}&{}-\frac{c}{D}&{}-\frac{1}{D}f_{N}\\ -\frac{1}{c}g_{V}&{}0&{}-\frac{1}{c}g_{N}\end{pmatrix}, \end{aligned}$$

where

$$\begin{aligned} f_{V}&=\Bigg [-\bar{g}_L-\bar{g}_{K}N_{\pm }-\frac{\bar{g}_{\text {Ca}}}{2\bar{v}_2}\left( 1-\tanh ^2\left( \frac{V_{\pm }-\bar{v}_1}{\bar{v}_2}\right) \right) (V_{\pm }-\bar{v}_{\text {Ca}}) \nonumber \\& -\frac{\bar{g}_\text {Ca}}{2}\left( 1+\tanh \left( \frac{V_{\pm }-\bar{v}_{1}}{\bar{v}_{2}}\right) \right) \Bigg ], \\ f_{N}&=-\bar{g}_K(V_{\pm }-\bar{v}_K), \\ g_{V}&=\frac{\psi }{2\bar{v}_4}\Bigg [ \Bigg \{\frac{1}{2}\left( 1+\tanh \left( \frac{V_{\pm }-\bar{v}_{3}}{\bar{v}_{4}}\right) \right) -N_{\pm }\Bigg \} \sinh \left( \frac{V_{\pm }-\bar{v}_{3}}{2\bar{v}_{4}}\right) \Bigg ]\nonumber \\ {}& +\frac{\psi }{2\bar{v}_4} \Bigg [\cosh \left( \frac{V_{\pm }-\bar{v}_{3}}{2\bar{v}_{4}}\right) \left( 1-\tanh ^2\left( \frac{V_{\pm }-\bar{v}_3}{\bar{v}_4}\right) \right) \Bigg ], \\ g_{N}&=-\psi \cosh \left( \frac{V_{\pm }-\bar{v}_{3}}{2\bar{v}_{4}}\right) . \end{aligned}$$

The eigenvalues of (19) are the solutions to the characteristic equation

20 $$\begin{aligned} \lambda ^{3}+P_{2}\lambda ^{2}+P_{1}\lambda +P_{0}=0, \end{aligned}$$

where

$$\begin{aligned} P_{2}=\frac{g_{N}D+c^{2}}{cD}, P_{1}=\frac{g_{N}+f_{V}}{D}, \text {and} P_{0}=\frac{f_{V}g_{N}-f_{N}g_{V}}{cD}. \end{aligned}$$
